# Technology, data, people, and partnerships in addressing unmet social needs within Medicaid Managed Care

**DOI:** 10.1186/s12913-024-10705-w

**Published:** 2024-03-23

**Authors:** Rachel Hogg-Graham, Allison M. Scott, Emily R. Clear, Elizabeth N. Riley, Teresa M. Waters

**Affiliations:** 1 Department of Health Management and Policy, College of Public Health, University of Kentucky, 111 Washington Ave, 107B, Lexington, KY USA; 2 Department of Communication, University of Kentucky, Lexington, KY USA; 3 Institute for Public and Preventive Health, Augusta University, Augusta, GA USA

**Keywords:** Social determinants of health, Medicaid, Managed care organizations, Health care organizations and systems

## Abstract

**Background:**

Individuals with unmet social needs experience adverse health outcomes and are subject to greater inequities in health and social outcomes. Given the high prevalence of unmet needs among Medicaid enrollees, many Medicaid managed care organizations (MCOs) are now screening enrollees for unmet social needs and connecting them to community-based organizations (CBOs) with knowledge and resources to address identified needs. The use of screening and referral technology and data sharing are often considered key components in programs integrating health and social services. Despite this emphasis on technology and data collection, research suggests substantial barriers exist in operationalizing effective systems.

**Methods:**

We used qualitative methods to examine cross-sector perspectives on the use of data and technology to facilitate MCO and CBO partnerships in Kentucky, a state with high Medicaid enrollment, to address enrollee social needs. We recruited participants through targeted sampling, and conducted 46 in-depth interviews with 26 representatives from all six Kentucky MCOs and 20 CBO leaders. Qualitative descriptive analysis, an inductive approach, was used to identify salient themes.

**Results:**

We found that MCOs and CBOs have differing levels of need for data, varying incentives for collecting and sharing data, and differing valuations of what data can or should do. Four themes emerged from interviewees’ descriptions of how they use data, including 1) to screen for patient needs, 2) to case manage, 3) to evaluate the effectiveness of programs, and 4) to partner with each other. Underlying these data use themes were areas of alignment between MCOs/CBOs, areas of incongruence, and areas of tension (both practical and ideological). The inability to interface with community partners for data privacy and ownership concerns contributes to division. Our findings suggest a disconnect between MCOs and CBOs regarding terms of their technology interfacing despite their shared mission of meeting the unmet social needs of enrollees.

**Conclusions:**

While data and technology can be used to identify enrollee needs and determine the most critical need, it is not sufficient in resolving challenges. People and relationships across sectors are vital in connecting enrollees with the community resources to resolve unmet needs.

**Supplementary Information:**

The online version contains supplementary material available at 10.1186/s12913-024-10705-w.

## Introduction

Individuals with unmet social needs, like food and housing insecurity and transportation challenges, experience higher rates of adverse health outcomes [1–7] and are subject to greater inequities in health and social outcomes [8]. Unmet social needs are especially prevalent among Medicaid enrollees [9]. For this reason, state Medicaid programs are particularly interested in testing strategies that encourage and incentivize Medicaid managed care organizations (MCOs) to identify and address the complex social needs of enrollees [10, 11]. Many Medicaid MCOs are now screening enrollees for their unmet social needs and connecting them to community-based organizations (CBOs) better equipped with knowledge and resources to address these needs [12, 13].

The use of screening and referral technology and data sharing are often considered key components in programs integrating health and social services to address social needs [12, 14]. Data sharing infrastructure has been highlighted as a way to streamline coordination and social need resolution [12, 14]. In some instances, successful integration has facilitated strong connections between health and social services organizations, ensuring that patients move efficiently between sectors [14–16]. Despite this emphasis on technology and data collection and some positive integration, research suggests substantial barriers exist in operationalizing effective systems [12, 17]. CBOs often have limited resources, financial and personnel, to put toward the use of advanced social need screening and referral systems [12, 17–19]. The reliance on grant funding and other time-limited resource streams likely presents another barrier in the adoption of tools [17]. CBOs can also be hesitant to adopt technology and data systems owned by MCOs, hospitals, and other clinically oriented organizations because of data privacy and HIPAA-related issues [16, 20].

Research examining health and community partnerships has identified technology adoption by CBOs and other social services organizations as an important barrier to collaboration [14, 15, 17]. Most prior studies examining data and technology include clinical organization perspectives on the use of tools but do not include robust information from community partners [12, 14, 16]. Further, those studies that do include perspectives from multiple organization types on the integration of health and social services are not focused on adopting screening and referral systems. Technology typically emerges in subthemes, and the evidence included does not provide in-depth information on benefits and challenges from both community and clinical partners [17].

This study examines CBO and MCO perspectives on the use of technology in social need screening and referral. The qualitative analysis presented here is part of a larger mixed methods study examining how Kentucky (KY) MCOs address unmet social needs in partnership with community organizations [21]. KY offers a unique opportunity to examine strategies addressing Medicaid enrollee needs. Just under 29% of all KY residents are enrolled in Medicaid, making it the third highest enrollment among US states [22]. KY is also geographically diverse, with distinct urban, rural, and Appalachian regions.

## Methods

### Setting and study population

A project Stakeholder Advisory Board (SAB), including representatives from all Medicaid MCOs, academia, a community-based organization, the State Department for Medicaid Services, and enrollees, met quarterly to provide expertise, guide research, and assist with the dissemination of study results. MCO representatives serving on our SAB were asked to 1) identify individuals in their organization leading efforts to address unmet social needs and population health outcomes among their enrollees and 2) identify CBOs they work closely with in their social need referral process. As part of a targeted sampling strategy, identified contacts were invited via email by the research team to participate in key informant interviews to discuss how MCOs and CBOs address social needs. Inclusion criteria were that participants were at least 18 years old, were employed at an MCO/CBO in Kentucky, and were willing to engage in an interview in English. A total of 32 MCO contacts were invited and 33 CBOs, giving us response rates of 81% and 58% respectively.

### Participants

Our sample of 46 participants comprised 26 representatives from 6 MCOs (ranging from 3 to 6 participants per MCO) and 20 representatives from 19 unique CBOs. MCO participants represented various organizational roles, including vice presidents, directors, population health, case management, and community engagement. CBO participants represented roles including directors, Chief Executive Officers, Chief Operating Officers, Medical Coordinators, Presidents, Chief Engagement officers, program managers, and outreach coordinators. The services provided by community-based organizations included food security, health, housing, employment, and work readiness, refugee and immigrant services, and community support; many CBOs addressed multiple social needs. CBO interviewees represented organizations operating in both urban and rural areas of the state.

### Data collection

In-depth one-on-one interviews with 46 stakeholders from identified CBOs (*n* = 20) and MCOs (*n* = 26) were conducted between May 24, 2021, and November 8, 2021. Interviews were conducted via Zoom, audio-recorded, and transcribed verbatim. The qualitative researcher and facilitator conducting these interviews have extensive training and experience with structural interviewing using a semi-structured interview guide. The guide used was developed for this study [23].

### Data analysis

We conducted an iterative content analysis of the transcribed interview data using qualitative descriptive analysis [24], an inductive, low-inference method designed to gain an accurate understanding of a phenomenon in the everyday terms of stakeholders. Our data analysis unfolded in two stages. The first stage involved open coding [25], in which the transcripts were independently coded by two authors and one study team member (AM, ER, and HS), who then met to discuss and reach consensus on the central themes in the data related to technology and data sharing. In this meeting, the authors identified the themes of to screen for patient needs, to case manage, to evaluate the effectiveness of programs, and to partner with each other. The second stage of analysis involved focused coding, with the three individuals again independently coding transcripts for subthemes within each identified central theme. The coders met again to compare findings and finalize themes (and subthemes for Theme 4). At this time, we recognized that there were areas of alignment, incongruence, and tension between the responses of participants from MCOs and CBOs, and we reached agreement in this meeting about which themes demonstrated each dynamic. Finally, all authors met a third time to review the subthemes and select illustrative quotations for each. All analytic decisions were made through discussion until consensus was reached. We used the team-based approach to reaching consensus, which considered dependability and trustworthiness of the data [26]. This paper focuses on responses addressing technology platforms and data sharing to support MCO and CBO partnerships.

## Results

We identified several themes related to the use of technology and data in MCO-CBO partnerships to address enrollee social needs. MCOs and CBOs noted differing levels of need for data, differing incentives for collecting and sharing data, and differing valuations of what data can or should do. MCO and CBO interviewees described how they collect and use data in their work, which fell into four major themes: to screen for patient needs, to case manage, to evaluate the effectiveness of programs, and to partner with each other. Within these themes, the interview responses illuminated areas of alignment between MCOs/CBOs, incongruence, and tension (both practical and ideological; see Table 1).

**Table 1:** Themes identified in interviews asking MCOs and CBOs about their purposes for and uses of data in programming

Theme	Areas of Alignment	Areas of Incongruence	Areas of Tension
**Collecting data on patient needs**	Usefulness: nearly all MCOs and CBOs recognized value in using data to identify patient needs and determine which needs to meet first	The degree to which this was done: all MCOs did this, only some CBOs formally used data to screen for needs; MCOs did this more frequently and comprehensively	Importance: MCOs highly valued data screening metrics for identifying patient needs. CBO responses had variability in the importance of using data to screen for needs
**Using data to support case management**	Usefulness: all MCOs and most CBOs saw value in data systems identifying resources available, tracking referrals and follow-ups, keeping notes, and staying in contact with patients	Data analytic capacity: variability in the sophistication of the data systems, such that most MCOs used data analytics to case manage (few CBOs did this)	Variety of data systems and platforms: MCOs and CBOs are invested in the system(s) they utilize but the systems do not communicate
**Using data to evaluate the effectiveness of programs**		Financial motivation: all MCOs use data to determine the financial impact/effectiveness of programs, only some CBOs relate data-driven evaluation to funding (usually to gain funding, not keep it)	Financial: CBOs’ lower level of enthusiasm for/ability to use data creates difficulty for MCOs when they rely on CBOs for data to justify their funding streams
		Ideological differences about whether data can be used to evaluate effectiveness: MCOs see data-driven evaluation as essential, but some CBOs feel that data hurts them in the metrics	Motivation for partnership: MCOs don’t want to work with CBOs on the basis of vision alone – they want to see evidence/data backing up the effectiveness of the program; CBOs feel pressure from MCOs to produce data to justify investment
		Capacity: CBOs may be more reluctant or less able to use data to evaluate programs due lack of resources (technological and workforce capacity)	
**Using data to partner with other MCOs and CBOs**	All MCO interviewees acknowledged that CBOs are doing good work, even if that cannot be quantified, and the ability to share that data is often related to CBO capacity and resources	Practical interfacing difficulty: MCO data systems tend to be more sophisticated than CBO data systems, and CBO systems don’t have the capacity to interface with MCO systems	Privacy and ownership concerns: MCOs ask CBOs to share data with them but MCOs don’t share data in return, CBOs highly value patient data confidentiality even from MCOs

### Theme 1. Alignment on collecting data to identify and prioritize patient needs

Using data to identify and prioritize patient needs was largely an area of alignment for MCOs and CBOs. All MCOs and nearly all CBOs recognized the value of data in this area. As one CBO noted,

*“By completing the needs assessment with our families, it helps the case managers understand your immediate needs.”*

Similarly, MCOs often used the data for targeted programming and social needs referrals,

“*When our members are enrolled, we attempt to engage them in our health risk assessment. And so that health risk assessment is going to not only ask them questions about their specific health, but also about some additional needs that would help us be able to identify them at enrollment and also to be able to target them for programs and other [benefits].”*

Several MCO and CBO interviewees also discussed using the data to understand individual enrollee/client needs and to track overall trends among their clients. As one MCO shared,

*“The end of 2021, we had a tremendous amount of referrals for food. And so maybe we need to look at doing some of our community investment work and partnering with additional providers and community partners that are in that space for next year.”*

There were some differences between MCOs and CBOs in the formality and degree to which social need data was collected. MCO interviewees, particularly those on the front lines of this work, could describe detailed and comprehensive data screening metrics for patient needs and how needs were tracked in their data systems. Using data on patient needs to identify areas for intervention was described as an essential part of patient care:

*“We use the screening data, not just to meet the individual member need, but to also inform health equity and types of programs that we bring to play...”*

CBO interviewees, on the other hand, had greater variability in their responses about the importance of using data on social needs at an organizational-level. Most described data as having potential value but stopped short of calling it essential for their operations. One CBO stated,

*“I don't know what I would do with the information if we had it.”*

Conversely, one food-oriented CBO reported that they collect demographic data and use that to help with distribution,

*“So think about the local pantry that I talked about earlier. Because we know, we drive a truck into [KY County]. We know that the last five times that we've been in [KY County], we saw, on average, 150 households at each of those five visits. That tells us how much product to put on the truck so that we don't run out.”*

### Theme 2. Differences in organizational capacity, mission, and resources influenced variability in data use to support case management

Using data to support case management activities was an area of both alignment and incongruence between MCOs and CBOs. All MCOs and many CBOs saw value in using data systems to identify resources available, track referrals and follow-ups, keep notes, and stay in contact with patients. However, there was considerable variability in the sophistication of the data systems. Most MCOs reported elaborate data tracking systems designed specifically for screening, referral, and tracking (e.g., combining medical records applications with Unite Us [27] or Find Help (formerly Aunt Bertha [28]). Some CBOs have systems designed specifically for tracking data (e.g., Electronic Health Systems or Vesta [29]), whereas others employ systems not designed specifically for tracking (e.g., Microsoft Excel spreadsheets). Most CBOs used informal data collection to screen for needs (e.g., Post-it notes, memory, a hand-written planner), and several CBOs reported that they did not use formal data systems to screen and track patient needs at all,

*“Are you kidding me? No books. What I usually tell anybody who's working with me is to either email me or text me, and that's my filing system.”*

MCO interviewees were more likely to report using data analytics to support and enhance case management. Frontline MCO workers spoke about this aspect of data use more often than executives, and many saw data systems as the answer to case management problems. As one MCO stated,

*“We do have a case management system that keeps track. So, we are able to schedule calls. They're able to pop back up on a calling queue, so that we're able to check in with members and attempt to continuously reach out to them. So, that's kind of how we try to make sure that those members don't fall through the cracks by continuously following up.”*

Most CBOs indicated that case management occurred but was more personalized and less attached to data and technology use,

*“We have a database that we use for client notes. We just record case notes in there. Some of our caseworkers keep basic Excel spreadsheets on their specific clients and what they're working on. Most of that would be informal.”*

Only one MCO specifically mentioned the limits of data systems for tracking and the need for a personal touch in case management, a perspective more in line with most CBO interviewees. The MCO shared this when discussing platform capabilities, stating,

*“We have a case management platform, of course, where we document everything, because just like everywhere else, if you don't write it down, it didn't happen, but a lot of it is just that manual follow-up and that human touch.”*

The variability in tracking system sophistication and capabilities between MCOs and CBOs was also frequently highlighted as one of the critical challenges in collaboration and a notable source of frustration for both sides. When discussing their partnerships with MCOs and data sharing, one CBO stated,

*“They really wanted to know about it. And so had to spend considerable time with them about, ‘This is what we do, this is how stuff works.’ And including it's like, ‘No, we can't track. We have no way of tracking [MCO] clientele through the [KY food security] program’."*

While MCO interviewees often noted this tension in collaboration, they were aware that capacity and resources typically made it harder for CBOs to track and collect data. One MCO interviewee noted,

*“I think the challenge is just the data piece and the complexity of the regulations that we have to navigate, all for good reason. When you're talking about how to best leverage those community resources, if we can't kind of have those data exchanges, it makes it so much more difficult. And so when you're trying to get at outcomes or have simplified referral processes, it just makes it harder because you may not be able to get through, they may not have the HIPAA, the high-tech clearance or whatever it is. It's expensive for them to have to do that.”*

### Theme 3. Funding and reimbursement structures shaped how MCOs and CBOs used data to evaluate program effectiveness

We found limited alignment between MCO and CBO perspectives on using data to evaluate social need programming and partnerships. Instead, evaluation was an area fraught with incongruencies and tension between the two sectors. The financial incentives and pressures for using data differ substantially between MCOs and CBOs. MCOs reported using data to evaluate the financial impact or effectiveness of programs (particularly claims data/utilization metrics) and partnerships to justify investments or show MCO executives that meeting unmet social needs is good business. As one MCO interviewee explained,

*“I think every anything that we’re doing with the community-based partner, we’re studying all that. We’re studying the reduction, so I’m able to say, okay, because we have this member in this [CBO program], in this residential treatment program, not only mama’s healthier, baby is not born exposed to opiates, no NICU, ER utilization down. I think that’s the neat thing, there’s your answer, right?”*

One reason MCOs seem to be driving data collection for demonstrated effectiveness/return on investment is that they are heavily regulated in terms of how they can invest funds,

*“We are doing payment innovation, we want to take money out of what’s being spent on health care and invest it into social services and that is not easy.”*

As another MCO highlighted continued investment often depends on what they can demonstrate,

*“Sometimes, there are finance guidelines, right? Like when I’m fighting for my budget, they’ll say, ‘Well, where’s the return on investment numbers?’.”*

Conversely, only a few CBOs used data-driven evaluation to support their financial operations. When CBOs did report using data for evaluation, it was typically in relation to using outcomes data in grant writing to gain funding specifically from MCOs, data which may not serve any other useful purpose for the CBO. As one CBO stated,

*“Another kind of pain point, and for like one of the managed care companies that we contract with, they give us $8,000 a year. But the requirements to receive that $8,000 is very data heavy. We have to go through and pull all this data, get different releases signed with the participants. It’s great to have extra money, but it’s also a lot of work and nothing really being tied to it, if that makes sense. They just want the data to be able to review and any good outcomes and success stories and stuff like that, which is great. But it’s a lot of work for not a lot of money.”*

### Theme 4. Tension in using data to partner with other MCOs and CBOs

Both MCO and CBO interviewees described several reasons why they engage in data sharing within MCO-CBO partnerships (e.g., to garner funding, demonstrate effectiveness, or enhance case management), even if the values and importance placed on data sharing differed between agency types. When data sharing existed or was being contemplated, interviewees still described several barriers to sharing, both practical and ideological.

Overwhelmingly, CBO interviewees expressed a perception that they had to report data to the MCOs to prove impact so MCOs would maintain the partnership or provide funding. The *first subtheme* revealed a notable *ideological difference between the MCOs/CBOs regarding whether data was useful to evaluate program effectiveness*. While data-driven evaluation is routine and relied upon by most MCOs, many CBO interviewees perceived that data and metrics could harm their operations, diverting time and energy from serving clients and that there is much about program effectiveness that simply cannot be captured using formal data tracking systems. When discussing the course of their partnerships with MCOs, one CBO highlighted,

*“So what does that support look like? Well, it is financial support for it. And, initially, it was very much focused on their clientele with [MCO] clientele and trying to track metrics about the impact that having access to better nutrition was going to have on the outcomes for their folks, right? So over the course of two years, I mean, we were able to show, "we," and I mean that collectively, we're able to show that it does have a positive impact. I mean, for [MCO], I think it's safe to say that they realize that it is more cost-effective to invest upfront in increasing access to healthy food better than the back end, to drugs and health care costs and all that kind of stuff. So they have, again, they have maintained that partnership.”*

Indeed, most MCOs expressed wanting data from their CBO partners to justify the relationship and a reluctance to build relationships if data capacity is not present. One MCO discussed this directly, stating,

*“They come us and they send us their flyer and they're like, "We want [MCO] to partner with us on our heart walk and we want you to give us $20,000." We still get a lot of people that do that because that's their old business model. Most of the time, we don't engage with those types of organizations. I always say, we want to hear from someone and I will take a meeting always if a community-based organization says, "We have an evidence-based solution that is solving for X," or "We have a solution that is solving for X and we want to work with you to help us prove that it's evidence-based," or we have research capabilities...”*

*Subtheme 2* illustrates how underlying the data sharing tension between CBOs and MCOs are *challenges related to the need for more effective and user-friendly interfacing between tracking and referral systems, as well as the limited capacity of CBOs to track and analyze data*. As mentioned, the sophistication of CBO data systems is highly variable, and even those organizations with more advanced tracking systems struggle with data sharing. When asked about data sharing, one CBO noted,

*“Well that's another pain point. In my history, in my experience, every health plan has their own data system that don't talk to one another, that are very convoluted and messy. Right now we're filling stuff in on an Excel spreadsheet.”*

Several MCOs also highlighted this as a challenge. As one MCO stated,

*“Our system is designed to deal with hospital systems and health care providers, there's many different levels. I mean we go through a pretty comprehensive system and you have to have all kinds of, meet all kinds of requirements, share data, and different pieces that for a small community-based organization providing housing services, they might not even have the capacity to meet those requirements.”*

Although some CBOs reported sharing data with MCOs willingly and saw this sharing as a natural facet of their partnership, other CBOs described significant *concerns about data privacy and ownership* (*subtheme 3*). They noted how important data privacy was to the clients they served and how their organization valued serving their clients without the need to collect personal data or share it. Some CBO interviewees indicated that sharing or even collecting private client data might compromise their ability to do their work and serve their clients well,

*“We respect their privacy, and we will never do any sharing of their data. In fact, a lot of people who come to us, one of the reasons they're with us is because we do not require them to show an ID.”*

*Subtheme 4* revealed how CBO and MCO interviewees expressed *concerns about relying on data and technology as the solution to social need screening and referral systems building*. Interviewees felt that data does not adequately capture utilization or partnership benefits. Primarily, this was attributed to issues related to data quality. One MCO interviewee highlighted this when discussing the challenges of understanding the quality of social need services:

*“We also don't have a really long track record of managing quality for this type of provider. We have very distinct report cards and quality cards for every hospital in the state of Kentucky. I can tell you what the outcomes for [Hospital 1] compared to [Hospital 2] and compared to [Hospital 3]. We have very clear metrics on those types of things. We do not have that for the sort of soft services, especially since we don't pay for them.”*

Most CBOs articulated challenges with data quality centered on their perception that data does not tell the whole story about what is happening at their organization and in the community. As one CBO noted,

“*We have a people problem. And I think right now there are a lot of hospitals and other organizations, MCOs, that want to kind of tech their way out of this. [T]hey're looking for technological [solutions] to try to streamline and expand services to folks. And that's just not really the answer. You need people.”*

MCO interviewees recognized that databases and their tracking systems may be limited in what they capture. In *subtheme 5*, several noted their *technological ability to comprehensively track organizations in a community as a significant limitation*. Maintaining accurate data has also been challenging because of community organization turnover and closures. As one MCO highlighted,

*“These national repositories don't have the local knowledge so they don't know the churches that do the hot meals and they don't know the small organizations that are getting up and off their feet and tied to this one or that one, or it's an offshoot of whatever. There are some smaller organizations that don't always get into those big directories and you don't always know about them unless you have boots on the ground, people who live and work in the community and actually know what those are.”*

Similarly, another MCO highlighted CBO data capacity as a major challenge in their partnerships, stating,

*“Biggest challenges. I guess, you could say data might be the challenges, to close the loop around the return on investment on some of these organizations that are not ... They just don't have the staffing, or the professional leadership, if you will, to do all the tracking. The ones that do, do it very well. The ones that don't, it's just that they don't have the resources.”*

In the final subtheme, all MCO interviewees acknowledged that *CBOs are doing good work*, even if that cannot be quantified, and the ability to share that data is often related to CBO capacity and resources. One MCO shared,

*“[Food Pantry CBO] who's just like [Named Female] and her husband [Named Male], they might be the greatest people and we might know that members like going there versus the other food bank because [Named Female] like bakes brownies and gives them a hug and we want to quantify that but also it's just not realistic because they don't have the infrastructure sometimes that's needed to prove the business case, solidify the partnership and ultimately inform policy.”*

## Discussion

Our study found alignment as well as discordance between MCOs and CBOs about how and when to leverage technology and data despite their shared mission to meet the unmet social needs of enrollees. Our findings offer important insights regarding why data and technology may create a barrier to effective MCO-CBO partnerships, potentially hindering efforts to improve health and social outcomes. They also provide guidance and identify key considerations for developing programs and partnerships that may be more effective in coordinating efforts between the two organizations.

As we observed in Themes 1 (Alignment on collecting data to identify and prioritize patient needs) and 2 (Differences in organizational capacity, mission, and resources influenced variability in data use to support case management), results suggest that data and technology can be important tools in screening and referral for social needs, but they are far from a universal panacea. Our data indicate that both logistical and cultural disconnects between MCOs and CBOs significantly limit data collection and sharing for coordination of services. On the logistical side, CBOs have extremely limited capacity (software, workforce) to collect and share data. Several participants reported serious concerns with collecting and sharing confidential client information. To make matters worse, MCOs use a range of proprietary and sophisticated referral and tracking systems that severely tax the resources and capacity of CBOs. On the cultural side, while MCOs view data and technology as essential to partnering with CBOs to meet enrollee social needs, CBOs do not. In fact, as we found in Theme 3 (Funding and reimbursement structures shaped how MCOs and CBOs used data to evaluate program effectiveness), many CBOs see data collection as a necessary evil to garner funding from potential donors. Instead, they emphasize the relationship-honoring aspects of their work as a core value.

Solutions that only focus on providing data collection and tracking technology to CBOs are unlikely to be completely successful because they fail to address the disparate cultures found in MCOs vs. CBOs. This conclusion is robustly supported by Theme 4 from our analysis (Tension in using data to partner with other MCOs and CBOs).In many ways, CBOs may view MCO efforts to grow their technological capacity as imposing profit-seeking values, norms, and structure rather than seeking true understanding and partnership. CBOs’ low enthusiasm for and capacity to use data can create difficulty for MCOs when MCOs rely on CBOs for data to justify their funding streams and partnerships. This fundamental disconnect is likely to severely impede partnership efforts without reevaluating the strengths and values each sector brings to the collaborative [30]. 

Successful partnerships are built on shared interest and trust [31]. Our study suggests a strong alignment between MCOs and CBOs in addressing the social needs of highly vulnerable Medicaid beneficiaries. This values alignment may offer a foundation for partnership. Our work underscores a key finding across studies on cross-sector partnerships integrating health and social services, more work must be done to build trust and understand each other’s organizational values [17, 19, 32]. MCOs and CBOs need each other to address social determinants of health (SDOH) effectively. MCOs have the resources and responsibility for finding more effective ways to support their beneficiaries. CBOs are ‘on the ground’ and have the trust of the clients they serve (many of whom are Medicaid enrollees). Forums that create a level playing field for both types of organizations and facilitate safe conversations to build trust are essential.

The Department of Health and Human Services (DHHS) has developed a three-pronged strategy for addressing SDOH: (1) better data, (2) improving health and social services connections, and (3) whole-of-government collaborations [8]. Our study suggests that their second strategy is essential and could be far more difficult than many imagine. Facilitating honest conversations about identifying and addressing the challenges in building these connections is a critical first step. Because many challenges involve “hearts and minds” and organizational culture, addressing these challenges will need to be a slow and iterative process. Moving forward, organizations like MCOs and other clinical partners must carefully consider how data and social need screening and referral technology can be a value-add to CBOs and not another burden on their already strained capacity.

## Limitations

While our sample included at least one representative from all six state MCOs and nineteen different CBOs, the generalizability of study results may not apply to other states. However, many of the MCOs in KY operate in national markets and often use similar strategies in different geographic areas. Insights likely shed light on similar efforts and challenges in other states and markets. Future studies examining the use of data and technology nationally in social need resolutions would provide confirmation of the results we present and any potential geographic variability. Additionally, participant perspectives may not necessarily represent their MCOs or CBOs. Finally, our cross-sectional view of technology and referral platforms provides a snapshot of current processes; a more in-depth longitudinal study would capture changes over time as technology constantly evolves.

## Conclusions

Despite a shared mission to meet unmet social needs, MCOs and CBOs do not agree on how and when to leverage technology and data. This discordance is a significant barrier to effective partnerships. Technology offers powerful tools for identifying and prioritizing enrollee needs and connecting them with services. However, trust and a shared understanding of organizational cultures and goals are critically needed to allow technology to realize its potential. Current efforts to build effective MCO-CBO partnerships should focus on creating a level playing field for all organizations and a space for honest conversations that can build strong connections and sustainable relationships across sectors.

### Supplementary Information

**Supplementary Material 1.**

## Data Availability

Deidentified aggregated data is available from the corresponding author (rachel.hogg@uky.edu) on reasonable request.
