# Supplementary material for: Technology, data, people, and partnerships in addressing unmet social needs within Medicaid Managed Care
Source: BMC Health Serv Res. 2024 Mar 23;24:368. doi: 10.1186/s12913-024-10705-w (PMC10960441; doi:10.1186/s12913-024-10705-w)
Supplement: Supplementary file 1 — Supplementary Material 1. [file 12913_2024_10705_MOESM1_ESM.docx]

**Consent Introduction:**

Investigators at the University of Kentucky are conducting a research project, supported by the Robert Wood Johnson Foundation, to study how Kentucky’s Medicaid managed care organizations are working with community-based organizations (CBOs) to address enrollees’ unmet social needs. As part of the study, we are talking to leaders at both Medicaid MCOs and CBOs to better understand their partnerships. We anticipate that our interviews will take 30-40 minutes.

Your participation in this project is completely voluntary. There are no penalties to you or your organization if you choose not to participate, or if you decide at any time to discontinue the interview. There is no real risks or benefits to your participation. Your responses are confidential, and we will not identify you or your responses in public materials without your express, written permission. The answers you give will be compiled into a summary report that we will share with you, but there is no compensation for your participation. We will also use summary results to prepare reports to our funder and one or more journal publications. If you have any questions about the research or your rights as a research subject, you can contact one of the study PIs, Dr. Rachel Hogg-Graham (859-xxx-xxxx) or Dr. Teresa Waters (859-xxx-xxxx).

By proceeding with the interview, you are giving your consent to participate in this study.

**Medicaid MCO Stakeholder/Leader Questions for Semi-Structured Interviews**

1. Can you tell me the story of how you got engaged in these efforts to address unmet patient needs? What do these efforts look like specifically at your organization?
2. How and when are Medicaid enrollees screened for unmet social needs?
3. How does your MCO prioritize efforts/services when enrollees have complex needs?
4. What Community Based Organizations (CBOs) does your MCO typically partner with to address unmet social needs?
5. How does your MCO select CBOs to partner with?
6. What factors are considered when selecting CBOs? Probe: for example, do you consider type of services offered, CBO service area/reach, CBO capacity (vs volume of services needed), documented quality/outcomes?
7. How does your MCO pay CBOs?
8. How does your MCO track referrals and share information with CBOs?
9. What has been most challenging in working with CBOs?
10. Are there any factors that have made particular CBO partnerships more successful?
11. What are you most proud of in your partnerships with CBOs?
12. Which specific organizational structures actually matter for addressing unmet needs? How can you tell when a program is mature enough to make a detectable impact on utilization?
13. How would you define “return on investment” for these efforts? Based on your definition, what has been the ROI for these efforts? What are other things you measure to assess the outcome of these efforts? (If they answer “reduced readmissions” or “blood glucose levels,” probe for other, perhaps more system-level outcomes.)
14. How do you address individual needs while also addressing community determinants of health (i.e., system-level issues)?
15. What has surprised you about working with CBOs/MCOs? What advice would you have for other MCO/CBO leaders in terms of working with CBOs/MCOs?

**Community Based Organization (CBO) Stakeholder/Leader Questions for Semi-Structured Interviews**

1. How does your organization prioritize efforts/services when enrollees have complex needs?
2. How do you decide which MCOs to partner with? What factors do you consider? Prompt: for example, adequate compensation, match to service area and service offerings, required reporting?
3. How are you paid by MCOs? Do you view this payment as adequate? What could be improved?
4. How do you track referrals from MCOs? Is there a mechanism for sharing information (to-from MCO)?
5. How do you ensure adequate capacity when agreeing to partner with an MCO?
6. What has been most challenging in working with MCOs?
7. Are there any factors that have made particular MCO partnerships more successful?
8. What are you most proud of in your partnerships with MCOs?

**Potential COVID questions to add**

1. How has COVID-19 impacted social needs in your enrollees?
2. Has your ability to provide services been impacted by COVID-19?
3. How has COVID affected your partnerships during COVID-19? (probe new ones, ones dissolving)
4. What of these changes may stick? What won’t? Why or why not?
